# Eye-Tracking Characteristics: Unveiling Trust Calibration States in Automated Supervisory Control Tasks

**DOI:** 10.3390/s24247946

**Published:** 2024-12-12

**Authors:** Keran Wang, Wenjun Hou, Huiwen Ma, Leyi Hong

**Affiliations:** 1 School of Intelligent Engineering and Automation, Beijing University of Posts and Telecommunications, No. 1 Nanfeng Road, Shahe Higher Education Park, Shahe Area, Changping District, Beijing 102206, China; evakr1206@163.com; 2 School of Digital Media & Design Arts, Beijing University of Posts and Telecommunications, No. 10 Xitucheng Road, Beijing 100876, China; 3 Beijing Key Laboratory of Network System and Network Culture, No. 10 Xitucheng Road, Beijing 100876, China; 4 Key Laboratory of Interactive Technology and Experience System, Ministry of Culture and Tourism, No. 10 Xitucheng Road, Beijing 100876, China

**Keywords:** automated supervisory control, trust measurement, trust calibration, eye tracking, Random Forest

## Abstract

Trust is a crucial human factor in automated supervisory control tasks. To attain appropriate reliance, the operator’s trust should be calibrated to reflect the system’s capabilities. This study utilized eye-tracking technology to explore novel approaches, given the intrusive, subjective, and sporadic characteristics of existing trust measurement methods. A real-world scenario of alarm state discrimination was simulated and used to collect eye-tracking data, real-time interaction data, system log data, and subjective trust scale values. In the data processing phase, a dynamic prediction model was hypothesized and verified to deduce and complete the absent scale data in the time series. Ultimately, through eye tracking, a discriminative regression model for trust calibration was developed using a two-layer Random Forest approach, showing effective performance. The findings indicate that this method may evaluate the trust calibration state of operators in human–agent collaborative teams within real-world settings, offering a novel approach to measuring trust calibration. Eye-tracking features, including saccade duration, fixation duration, and the saccade–fixation ratio, significantly impact the assessment of trust calibration status.

## 1. Introduction

Automated supervisory control tasks often involve high-risk decision making, particularly in the aeronautical-military domain [1,2], characterized by quickly changing mission scenarios, time-sensitive information, and significant uncertainty and openness. The human operator typically performs as a supervisor [3], rapidly processing complex, multidimensional information and collaborating with automation-integrated intelligent agents to enhance decision-making outcomes in joint surveillance scenarios. Trust, defined as “the attitude that an agent will help achieve an individual’s goals in a situation characterized by uncertainty and vulnerability”, is essential in shaping human–agent interaction in this context [4]. On one hand, a lack of trust compels operators to invest additional effort in verifying the accuracy of information provided by agents [5], potentially leading to decreased decision-making performance in team tasks and even the neglect or misjudgment of critical information [6]. On the other hand, excessive trust may reduce operators’ vigilance in active monitoring [7], thereby increasing unknown risks and potentially causing safety incidents. Therefore, real-time assessment of operators’ trust calibration status—ensuring that perceived trust aligns with the actual capabilities and reliability of the agent [8]—and implementing strategic adjustments when operators exhibit over-trust or under-trust are crucial. These measures are essential for fostering higher-quality interactions between operators and intelligent agents, improving performance in situational supervisory control tasks, and mitigating human–agent conflicts arising from trust biases [9,10].

Accurately measuring trust in automated systems is essential for trust calibration. Researchers have proposed various methods for assessing trust. (1) Self-report scales: These are the most commonly used tools for measuring user trust due to their intuitive nature, cost-effectiveness, and ease of use [11,12]. Self-report scales reliably capture users’ perceived trust at a specific point in time, often at the conclusion of an experiment. However, they do not provide continuous measurements and are therefore unable to capture real-time trust fluctuations. Additionally, the data collection process often requires repeated interruptions during tasks, which can disrupt operators performing high-risk decision-making tasks. (2) Behavioral measurements: This approach objectively measures trust by observing and recording behavioral indicators during continuous tasks, such as acceptance of agent recommendations [13] or task performance [14]. Some studies combine behavioral measurements with self-reports [15,16,17] to advance quantitative trust assessment. However, behavioral indicators are often derived from specific scenarios and tasks, making it challenging to generalize their application to situational supervisory control tasks. (3) Physiological and neurophysiological measurements: These methods offer high temporal resolution, providing an alternative for real-time trust measurement [18,19]. Techniques include eye-gaze tracking [20], electrodermal activity (EDA), electroencephalography (EEG) [21], and emotional representations from voice data [22]. While these methods excel in capturing continuous data, they often struggle to explain the underlying mechanisms linking the indicators to trust. Additionally, they typically require complex and intrusive data collection equipment, which can be impractical in many applications.

In real situational supervisory control environments, operators’ prominent behavioral actions involve continuously adjusting their attention allocation under high time pressure to execute high-risk decisions [23]. Evidence suggests that eye movements can intuitively reveal the context of users’ activities and their cognitive processes [24]. Specific eye-tracking metrics have shown sensitivity to human trust [20], and eye-tracking devices are relatively simple to set up, causing minimal interference. This offers a promising, non-intrusive approach to achieving real-time trust monitoring. However, establishing a direct mapping between eye-tracking signals and the dynamics of operators’ trust remains a complex challenge. To address this, this study aims to develop a classification and regression model based on eye-tracking data and self-report scales using machine learning algorithms. The model is designed to efficiently and cost-effectively identify operators’ trust states in automated situational supervisory control tasks and is validated through simulated experiments. This research seeks to provide a novel approach for continuous, real time, and non-intrusive trust state measurement.

## 2. Background

This study follows Lee and See’s [5] description of trust as “the attitude that an agent will help achieve personal goals in situations filled with uncertainty and vulnerability”. This definition of trust is by far the most widely used in subsequent research on trust in automation [19]. Under this definition, trust is viewed as internal information within humans, which is not directly observable [10], posing significant challenges for trust measurement. Trust is not static; it evolves dynamically over time through repeated direct and indirect interactions within a team. Increasing users’ trust is insufficient; attention must also be given to the entire continuum of trust, which includes over-trust, calibrated trust, under-trust, misunderstanding, and distrust [6]. Some studies in the literature propose terms like calibration points [25] or critical states [26] to classify the situations when the intervention for calibrating trust is needed. In this study, calibrated trust is trust [that] matches the true capabilities of the automation [5], other cases lead to over-trust or under-trust. Terms like appropriate trust, calibrated trust, and appropriate reliance have often been used interchangeably in recent research [27,28,29].

Trust is a continuous and dynamic psychological process [19], and self-report scales are the most intuitive tools for measuring users’ psychological states. However, since self-report scales cannot provide continuous measurements, they can only capture trust levels at discrete time points. Addressing how to obtain more continuous and accurate trust dynamics is a key focus of this study. Some researchers have attempted to infer trust from the perspective of its formation and evolution. For example, Xu and Dudek [30] developed an online probabilistic trust inference model based on a dynamic Bayesian network framework, treating human trust in agents as a hidden variable that can be estimated by analyzing automation performance and agent behaviors. Similarly, Yaohui Guo [31] simulated the trust dynamics between humans and robotic agents over time, proposing a personalized trust prediction model. These mathematical models align with empirical findings on the characteristics of trust dynamics: (1) Trust at a given moment is significantly influenced by trust from the preceding moment [32]; (2) negative feedback loops with automated agents have a stronger impact on trust adjustment than positive feedback loops [32,33]; and (3) human trust tends to stabilize during repeated interactions with the same autonomous agent [34]. These studies provide a theoretical foundation and empirical insights for constructing mathematical models based on trust dynamics during actual interactions. By leveraging such models, it becomes possible to infer and fill the temporal gaps left by self-report scales, thereby enabling a more comprehensive understanding of trust dynamics.

Preliminary research has shown that raw eye-tracking data can be used to calculate various eye-tracking metrics, which have been successfully employed in past studies to infer diverse perceptual and cognitive challenges [35,36]. However, empirical evidence regarding the feasibility and validity of using eye-tracking data to infer human trust in automation remains limited. Some studies have explored the diagnostic role of a few eye-tracking metrics in specific scenarios. For instance, Lu and Sarter [20] proposed using eye-tracking metrics, such as fixation duration and saccade path length, to infer real-time trust in humans. Hergeth [37] found that gaze behavior provides a more direct measurement of trust in automation compared to other behavioral measures. Similarly, Gold C [38] demonstrated a significant negative correlation between subjective trust ratings and operators’ monitoring frequency. Moreover, combining basic metrics into composite indicators [39,40] for measuring visual attention distribution offers an additional approach to assess operators’ trust states more comprehensively.

In terms of algorithm selection, Lu [36] discussed the feasibility of modeling eye-tracking data using three machine learning techniques—logistic regression, k-nearest neighbors (kNN), and Random Forest—as well as two deep learning methods, MLP and CNN. The study also examined the accuracy of modeling at both individual and group levels. In practical applications, advanced deep learning models such as CNNs [41] often require significant computational resources and large datasets, which can limit their real-time applicability. Furthermore, interpretability is another critical factor influencing the adoption of these technologies. Addressing challenges such as imbalanced class distribution in training data [42] also remains an ongoing issue. These discussions provide valuable insights and references for selecting algorithmic models in this study.

## 3. Materials and Methods

This section focuses on detailing the participants, experimental materials, experimental tasks, data collection, machine learning models, and model evaluation. Each component is critical to the study’s methodology.

### 3.1. Participants

A total of 12 participants were recruited from the university for this experiment. Due to interruptions during the experiment or eye tracker malfunctions resulting in incomplete data, the data from two participants were excluded. The final analysis included data from 10 participants, aged between 19 and 28 years (M = 23.2, SD = 2.6), with 5 males and 5 females. All participants possessed theoretical knowledge of automated supervisory control tasks, with 8 of them having substantial practical experience. All participants had normal or corrected-to-normal vision and no color vision deficiencies. Written informed consent was obtained from all participants prior to data collection, and they were compensated for their participation.

### 3.2. Experimental Materials

**Figure 1:**
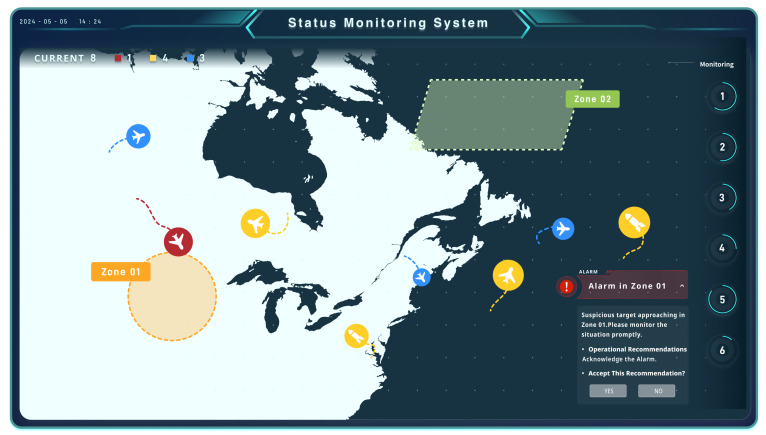
This is the experimental material interface at a specific moment, featuring moving targets (aircraft, fire-fighting apparatus) and hazard zones. An alert pops up in the bottom right corner when targets approach the hazard zones.

A simulated system was developed specifically for this study to emulate a real-world automated supervisory control task (see Figure 1). Various targets, such as aircraft and firepower equipment, appeared and disappeared at random positions on the system’s situational awareness map, following a complex yet interpretable pattern. These targets moved along predetermined paths. When the intelligent agent detected suspicious targets approaching or entering key focus areas, a popup alert would appear in the bottom-right corner of the interface.

To avoid excessive cognitive load, the situational map displayed a maximum of 10 targets at any given time. Additionally, no consecutive alerts occurred within a 5 s interval to minimize overlapping popup windows, which could interfere with data collection.

The monitoring outcomes of the automated system could fall into four categories: hit, miss, false alarm, and correct rejection (see Table 1). The reliability of the agent was defined as

(1) $$\mathrm{Reliability}=\frac{\mathrm{Number}\;\mathrm{of}\;\mathrm{Hits}+\mathrm{Number}\;\mathrm{of}\;\mathrm{Correct}\;\mathrm{Rejections}}{\mathrm{Total}\;\mathrm{Number}\;\mathrm{of}\;\mathrm{Tasks}}.$$

This study configured the intelligent agent in the system to operate at four predefined reliability levels: 50%, 70%, 80%, and 90%. However, due to the randomness inherent in the experimental process, the actual reliability of the agent was calculated based on the system’s backend logs after the experiment.

### 3.3. Experimental Tasks

Before the experiment, participants underwent a 10-minute practice session to familiarize themselves with the interface and interaction process of the simulation system. During this session, they were given a walk-through of the experimental procedure to ensure they fully understood the objectives and steps. This practice aimed to minimize the influence of individual differences, such as familiarity with the interface layout or operational habits, on the experimental results.

In the formal experimental phase, participants were instructed to supervise all appearing and disappearing targets as closely as possible. When a suspicious target approached or suddenly entered a key focus area, participants had to decide whether to accept the system’s automated alert by selecting “yes” or “no” in the popup warning. This decision could be based on the situational status or their own theoretical knowledge and experience:

- Selecting “yes” prompted the system to log the alert details in the backend.
- Selecting “no” caused no system response but still logged the decision in the backend.

Each participant performed 30 continuous state discrimination tasks under each of the four system reliability levels (50%, 70%, 80%, and 90%). After every six tasks, participants completed an interim trust scale to assess their trust level at that point.

The experiment lasted approximately 40 min for each participant. Before each session, participants were reminded of the urgency and rigor required for the tasks and were informed of the system’s reliability level for that session.

### 3.4. Data Collection

**Figure 2:**
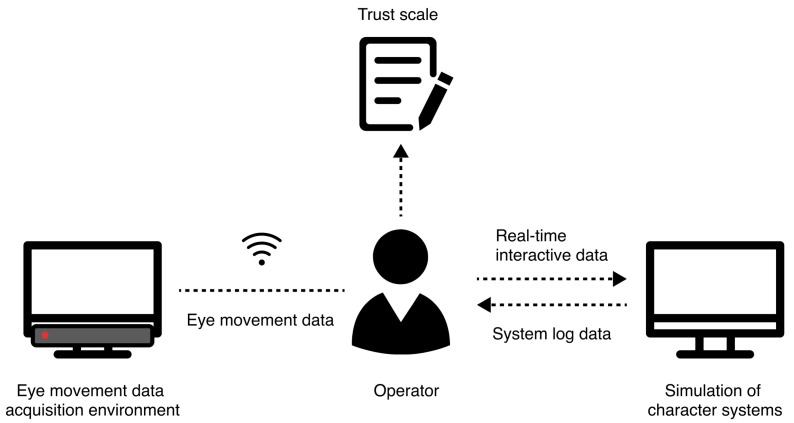
Human–agent trust model experimental scenario construction.

Eye-tracking data were collected using the Tobii Pro Fusion eye tracker in conjunction with the Tobii Pro Lab version 1.241 software [43], with a sampling rate of 250 Hz. A Windows 10 laptop was used for data recording and storage. Additionally, screen recordings of the experimental sessions were captured using the commercial software Bandicam version 7.1.0 [44]. The data collection setup is illustrated in Figure 2.

The data collected in this experiment included both objective and subjective measures, as detailed below:

A. Real-time interaction data

Real-time interaction data were gathered by observing the participants’ decisions to select “yes” or “no” in response to the automated agent’s alerts. These data were directly extracted from the screen recordings of the experimental sessions.

B.

System log data

System log data captured the performance of the automated alert system, including the four possible outcomes: hit, miss, false alarm, and correct rejection.

C.

Self-report scale data

Subjective data were obtained from participants’ responses to a staged self-report trust scale administered during the experiment. The scale used was a derivative version of a validated authoritative scale [12], adapted for greater precision and relevance to participants’ perceptions of the specific system used in this study.

To avoid misunderstandings due to language differences, the wording of the original items was revised appropriately to ensure a comprehensive exploration of perceived trust. Each item was rated on a 7-point Likert scale (1 = strongly disagree, 7 = strongly agree). Higher aggregate scores indicated higher levels of trust in the system. The scale’s reliability and validity were tested, and the raw scores were normalized and standardized to a 100-point scale. The detailed scale items are provided in Appendix A.

D.

Eye-tracking data

The eye tracker recorded various eye movement behaviors, such as fixations, saccades, and smooth pursuits, which collectively formed eye-tracking segments. To ensure comprehensive data capture, the Tobii Pro Fusion eye tracker recorded the entire experiment, including unrelated segments such as task interruptions and questionnaire completion. These irrelevant segments were trimmed using the Tobii Pro Lab software [43], resulting in two types of eye-tracking datasets:

- Raw eye-tracking data: This dataset included only raw fixation point coordinates represented as (x, y) pairs. Before inputting the data for analysis, preprocessing steps were applied, including handling missing data, down-sampling, and normalization. Missing data points were filled using the value from the preceding data point.
- Feature-extracted data: This dataset contained extracted features based on three primary eye-tracking metrics: pupil diameter, fixation, and saccade characteristics. These features were used to analyze participants’ attention and trust-related behaviors.

Table 2 presents the structure of the processed data. No. represents the task code, with each sequence consisting of six tasks, repeated five times by each participant. Interaction data indicate whether the participant accepted the system’s suggestion, with two possible states: yes or no. System data represent the relationship between the automated system’s detection results and the actual outcomes. Scale data and eye-tracking data describe the forms in which these two types of data were collected.

### 3.5. Trust State Classification and Regression Model Based on Two-Layer Random Forest

Based on the review in Section 2 (Background), the selected algorithm must meet the following criteria. (1) High interpretability: The model should offer strong interpretability to ensure that the relationship between input features (e.g., eye-tracking metrics) and trust states can be clearly understood and communicated. (2) Low resource usage: The algorithm should not require excessive computational resources, enabling its implementation in real-time systems without significant delays. (3) Robustness to data imbalance: The model should effectively handle imbalanced data distributions in the dataset to prevent biased predictions and ensure reliable performance across different trust states.

Random Forest [45] is a classic ensemble learning algorithm suitable for both classification and regression tasks in machine learning. The two-layer Random Forest model was chosen because it meets these requirements, achieving a balance between interpretability, computational efficiency, and adaptability to data imbalance through its ensemble learning approach and feature selection mechanisms. Additionally, the model leverages the hierarchical nature of the algorithm to enhance detection accuracy.

(1) First Layer: Random Forest Classification Model

The first layer consists of a Random Forest classification model built from multiple decision trees. These trees are trained following two fundamental principles: feature randomness and data randomness. Each decision tree uses a random subset of the raw eye-tracking data samples for training, ensuring robustness against overfitting and enhancing the model’s generalization ability.

As a preliminary screening model, the first layer prioritizes the detection of significant deviations in trust levels, specifically over-trust and under-trust relative to calibrated trust. Additionally, it determines the importance ranking of each eye-tracking feature, providing crucial information for the second-layer training process and ensuring that the most relevant features are emphasized in subsequent analysis.

(2) Second Layer: Random Forest Regression Model

The second layer focuses on the most critical features identified in the first layer to enhance detection performance. This layer is trained on a refined feature set that includes the highest ranking features from the first layer, along with newly introduced metrics as input features for the regression model.

The training process constructs decision trees that leverage this curated feature set to improve accuracy and robustness. For classification tasks, the ensemble decision is made through a majority voting mechanism, where the influence of individual incorrect predictions on the final classification is minimized. For regression tasks, the results of all decision trees are averaged to produce the final regression output. Each tree contributes equally to the final outcome, and the importance of the number of weak learners (trees) in the model is secondary to the performance of individual weak learners.

**Figure 3:**
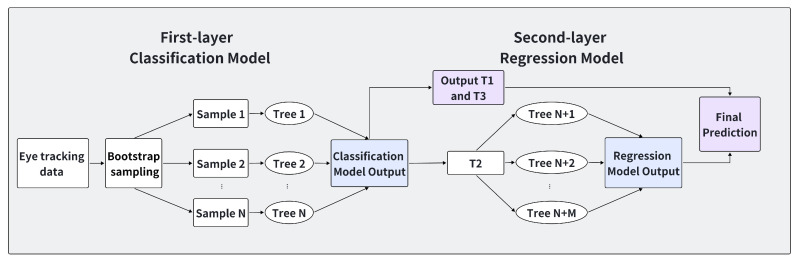
The architecture of the double-layer Random Forest model.

The hierarchical structure of the two-layer model enhances both robustness and accuracy, providing a scalable solution for trust state detection. Figure 3 illustrates the architecture of the dual-layer Random Forest model, outlining the training process and hierarchical structure. Both parts of the Random Forest model were implemented using the standard tools provided in the Python library Scikit-learn [46].

### 3.6. Model Evaluation

Various metrics are used to comprehensively evaluate the performance of the two-layer Random Forest model.

#### 3.6.1. Confusion Matrix

The confusion matrix [47] is a commonly used tool for evaluating classification problems. For a K-class classification problem, it represents the classifier’s prediction results in a k × k matrix format. In binary classification, samples are categorized based on the combination of their true outcomes and the model’s predicted outcomes into four categories:

- True positive (TP): The model correctly predicts a positive instance.
- True negative (TN): The model correctly predicts a negative instance.
- False positive (FP): The model incorrectly predicts a negative instance as positive.
- False negative (FN): The model incorrectly predicts a positive instance as negative.

The first letter indicates whether the prediction is true (T) or false (F), while the second letter denotes the classifier’s decision: positive (P) for a positive prediction and negative (N) for a negative prediction. Table 3 illustrates the types of combinations between actual and predicted outcomes.

This structure allows for detailed analysis of the model’s performance, highlighting areas such as precision, recall, and overall classification accuracy.

#### 3.6.2. First-Layer Random Forest Classification Model Evaluation

The first layer of the Random Forest model is a multi-class classification model with three categories. The primary metrics for evaluating the model’s effectiveness are accuracy, precision, and recall [48]:

Definitions:

1. Accuracy: The proportion of correctly predicted instances out of the total instances. It evaluates the model’s overall classification performance.
  (2) $$\mathrm{Accuracy}=\frac{\mathrm{TP}+\mathrm{TN}}{\mathrm{TP}+\mathrm{FP}+\mathrm{TN}+\mathrm{FN}}.$$
2. Precision: The proportion of true positive predictions among all positive predictions made by the model. A higher precision indicates stronger ability to distinguish negative samples.
  (3) $$\mathrm{Precision}=\frac{\mathrm{TP}}{\mathrm{TP}+\mathrm{FP}}.$$
3. Recall: The proportion of actual positive samples that were correctly predicted by the model. A higher recall indicates stronger ability to detect positive samples.
  (4) $$\mathrm{Recall}=\frac{\mathrm{TP}}{\mathrm{TP}+\mathrm{FN}}.$$

#### 3.6.3. Second-Layer Random Forest Regression Model Evaluation

The second layer of the Random Forest model addresses a regression task. The following common metrics were used to evaluate the model’s accuracy and overall performance:

1. RSS, MSE, RMSE, and MAE provide measures of accuracy, indicating how closely the model’s predictions align with the actual data.
  - RSS (residual sum of squares): Measures the total squared difference between predicted and actual values.
    (5) $$\mathrm{RSS}=\sum_{i=1}^{n}{\left(y_{i}-{\hat{y}}_{i}\right)}^{2}.$$
    where $y_{i}$ is the actual value, ${\hat{y}}_{i}$ is the predicted value, and *n* is the number of observations.
  - MSE (mean squared error): The average of the squared differences between predicted and actual values. It penalizes larger errors more heavily.
    (6) $$\mathrm{MSE}=\frac{1}{n}\sum_{i=1}^{n}{\left(y_{i}-{\hat{y}}_{i}\right)}^{2}.$$
  - RMSE (root mean squared error): The square root of MSE, providing an interpretable error measure in the same units as the target variable.
    (7) $$\mathrm{RMSE}=\sqrt{\mathrm{MSE}}.$$
  - MAE (mean absolute error): The average of the absolute differences between predicted and actual values. It gives equal weight to all errors.
    (8) $$\mathrm{MAE}=\frac{1}{n}\sum_{i=1}^{n}\left|y_{i}-{\hat{y}}_{i}\right|.$$
2. **$R^{2}$** reflects the model’s ability to explain the variance in the data comprehensively. An $R^{2}$ value closer to 1 indicates better performance, signifying that the model captures more information from the actual labels.
  - $R^{2}$ (coefficient of determination): Measures the proportion of the variance in the dependent variable that is predictable from the independent variables. It evaluates the model’s ability to capture the variability in the data,
    (9) $$R^{2}=1-\frac{\mathrm{RSS}}{\mathrm{TSS}}.$$
    where TSS is the total sum of squares:
    (10) $$\mathrm{TSS}=\sum_{i=1}^{n}{\left(y_{i}-\bar{y}\right)}^{2}.$$

## 4. Data Computation and Analysis

This section provides a detailed explanation of the construction and computation of the dynamic trust mathematical model, trust state annotation, feature engineering, and hyperparameter tuning of the classifier. Each step plays a vital role in building a robust framework for trust calibration and enhancing the model’s performance.

### 4.1. Construction and Computation of the Dynamic Trust Mathematical Model

Since self-report scales cannot provide continuous measurements, they only capture trust states at discrete time points. A key focus of this study is to obtain a more continuous and accurate representation of dynamic trust as a mapping target for eye-tracking data. Based on the review in Section 2 (Background), constructing a mathematical model of trust dynamics from actual interaction processes and using it to infer the temporal gaps in self-report scale data is theoretically feasible.

This section draws on the approach by Guo et al. [31], employing the mathematical expectation of a Beta distribution with performance-inducing parameters to model dynamic trust in the context of human–agent interactions during supervisory control tasks. The Beta distribution is particularly sensitive to the relationship between the mean and standard deviation, offering high interpretability, which makes it well suited for measuring the error between predicted and actual trust values [49].

After the agent completes the *i*th task, i=1,2,⋯, the human agent’s temporal trust follows a Beta distribution:

(11) $$t_{i}∼Beta\left(\alpha_{i},\beta_{i}\right).$$

The predicted trust ${\hat{t}}_{i}$ is calculated by the mathematical expectation of $t_{i}$:

(12) $${\hat{t}}_{i}=E\left(t_{i}\right)=\frac{\alpha_{i}}{\alpha_{i}+\beta_{i}}.$$

The parameters $\alpha_{i}$ and $\beta_{i}$ are the defining parameters of the Beta distribution, and their update rules adhere to the principles of trust formation and evolution in human–agent interactions. (1) Influence of prior trust: Trust at the current time step is significantly influenced by the trust level at the previous time step [32]. (2) Negative feedback loops have a greater impact on trust adjustment compared to positive feedback loops [32,33]. (3) Stabilization through repeated interaction: Human trust tends to stabilize during repeated interactions with the same automated agent [34].

(13) $$\begin{array}{cc} \alpha_{i} & =\left\{\begin{array}{cc} \alpha_{i-1}+w^{s}, & \mathrm{if}\;r_{i}=1,\\ \alpha_{i-1}, & \mathrm{if}\;r_{i}=0, \end{array}\right.\\ \beta_{i} & =\left\{\begin{array}{cc} \beta_{i-1}+w^{f}, & \mathrm{if}\;r_{i}=0,\\ \beta_{i-1}, & \mathrm{if}\;r_{i}=1. \end{array}\right. \end{array}$$

The variable $r_{i}$ represents the interaction result of the human–agent team during the *i*th task and can be determined by comparing the participant’s real-time interaction data with the system’s log data. When a positive feedback loop occurs (i.e., the participant observes that the monitoring intelligent agent issued a correct alert), $r_{i}=1$. When a negative feedback loop occurs (i.e., the participant observes that the monitoring intelligent agent failed to issue a correct alert), $r_{i}=0$. $w_{s}$ and $w_{f}$ are the gains due to the human agent’s positive and negative experiences with the agent. The superscript *s* stands for success and *f* stands for failure.

After *n* tasks, the human–agent team accomplishes $n^{s}$ tasks and fails $n^{f}$ tasks. Then,

(14) $$t_{i}∼Beta\left(\alpha_{0}+n^{s}w^{s},\beta_{0}+n^{f}w^{f}\right).$$

To infer the model’s parameters, after the *m*th task, m=1,2,⋯, and given the human–agent interaction result $R_{i}=\left\{r_{1},r_{2},\cdots ,r_{m}\right\}$, this study determines trust $T_{i}=\left\{t_{1},t_{2},\cdots ,t_{m}\right\}$ by the parameter set $\theta =\{\alpha_{0},\beta_{0},w_{s},w_{f}\}$. θ is updated with each new interaction between the human and the agent. Specifically, θ can be inferred from the trust value of the previous task and the interaction outcome of the current task.

The true value of the parameter θ is estimated as the value that maximizes the probability distribution P(θ), making it the maximum likelihood estimation (MLE) of the parameter:

(15) $$\begin{array}{cc} \theta & =\underset{\theta }{\mathrm{argmax}}P\left(T|\theta ,R\right),\\ & =\underset{\theta }{\mathrm{argmax}}\prod_{i=1}^{n}Beta\left(t_{i};\alpha_{i},\beta_{i}\right). \end{array}$$

where $\alpha_{i}$ and $\beta_{i}$ are determined by Equation (13).

**Figure 4:**
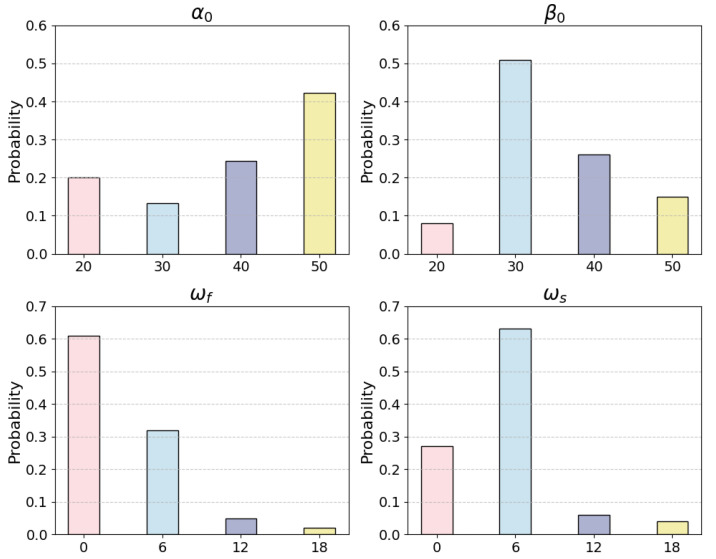
Prior distribution graphs of four parameters.

Using the data with a reliability level of 90% as an example, the probability P(θ) can be estimated using Equation (15). Figure 4 demonstrates the prior distributions of the four parameters learned through maximum likelihood estimation (MLE).

**Figure 5:**
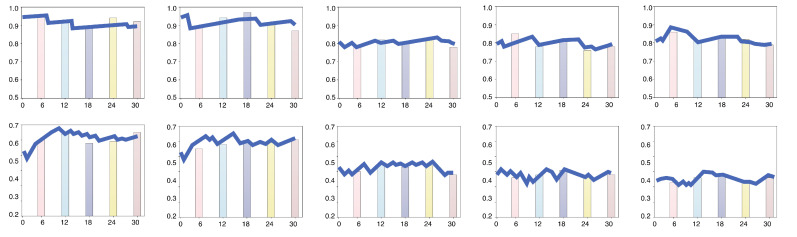
Prediction results of trust levels for 10 participants.

The proposed model successfully captured the trust dynamics of many participants, achieving high-quality data fitting. Figure 5 illustrates the prediction results for 10 participants, with the horizontal axis representing the task sequence and the vertical axis representing the trust level. The bar chart displays the trust values collected from the staged self-report questionnaires (scaled to two decimal places for computational convenience), while the line graph represents the trust levels predicted by the mathematical model. This visualization highlights the alignment between the subjective trust scores and the model’s predictions, demonstrating the effectiveness of the proposed approach in estimating dynamic trust states.

The root mean squared error (RMSE) was used to measure the deviation between the predicted values and the actual self-report scores. A smaller RMSE indicates that the predicted values are closer to the actual values, reflecting higher precision of the predictive model. In this study, the RMSE was calculated using the Python library Scikit-learn [46], and the result was 0.0521, demonstrating the good predictive performance of the model. Additionally, the trust dynamics prediction model exhibited varying performance under different system reliability levels (50%, 70%, 80%), as shown in Table 4. However, overall, the model maintained a balanced performance across these reliability levels, further validating its robustness and adaptability.

### 4.2. Trust State Annotation

Based on the research reviewed in Section 2 (Background), calibrated trust is trust [that] matches the true capabilities of the automation [5]; other cases lead to over-trust or under-trust. Since perceived trust and system capability are measured on different scales, comparing them is a challenge.

This section uses the model described in Section 4.1 to fill in the missing data from the self-report trust scales over time. Through normalization, the user’s perceived trust scale s is expressed with a score ranging from 0 to 100. At the same time, this study measure the real reliability r of the intelligent agent based on four possible outcomes (hit, miss, false alarm, and correct rejection). The reliability r is calculated as the ratio of the number of hits and correct rejections to the total number of tasks, which is then transformed into a score ranging from 0 to 100. By comparing the mathematical relationship between s (perceived trust) and r (system reliability), the trust state can be classified into three categories:

(16) $$\left\{\begin{array}{c} \mathrm{under}-\mathrm{trust}:s<r,\\ \mathrm{calibrated}\;\mathrm{trust}:s=r,\\ \mathrm{over}-\mathrm{trust}:s>r. \end{array}\right.$$

Defining intermediate trust states is challenging. To minimize the impact of imbalanced categories [42] on model accuracy and reduce noise, the distribution of data across trust categories was carefully analyzed. The boundaries for calibrated trust were expanded to include more data under this label. As a result, trust states were classified into the following categories:

(17) $$\left\{\begin{array}{c} T1:\mathrm{under}-\mathrm{trust}:\;s<0.95r,\\ T2:\mathrm{calibrated}\;\mathrm{trust}:\;0.95r\leq s\leq 1.05r,\\ T3:\mathrm{over}-\mathrm{trust}:\;s>1.05r. \end{array}\right.$$

### 4.3. Feature Engineering

Due to the large number of eye-tracking metrics, extracting, transforming, and selecting the most valuable features is essential to improve model accuracy and enhance its generalization ability. These steps enable the model to better capture patterns within the data. Based on the studies reviewed in Section 2 (Background), preliminary research has revealed that raw eye-tracking data can be used to compute a variety of metrics. These metrics have been successfully applied in previous studies to infer various perceptual and cognitive challenges, including fixation, saccade, and pupil diameter, among others.

In this section, Spearman correlation coefficients [50] are used to examine the relationships between the three trust states annotated in Section 4.2 and the statistical metrics derived from the corresponding eye-tracking segments. The Spearman coefficient measures the direction and strength of a monotonic relationship between two variables. The correlations were calculated using the SPSS version 25.0 software [51], and the analysis results are summarized in Table 5.

Although correlation does not imply causation, the results indicate the following:

1. Most eye-tracking metrics can be used to evaluate human–machine trust states.
2. Metrics such as fixation index, saccade duration, fixation–saccade time ratio, and pupil diameter change rate show a very strong correlation with human–agent trust states.

### 4.4. Hyperparameter Configuration for the Classifier

(1) Hyperparameter Selection for the First-Layer Random Forest Classification Model

The main goal at this stage is to distinguish between the T1 (under-trust) and T3 (over-trust) trust states. The eye-tracking data classified into the T2 (ambiguous trust) state is processed separately. Therefore, the overall accuracy of the model does not fully reflect the classification objective. In this section, the precision of the T1 (under-trust) and T3 (over-trust) segments is used as the criterion for selecting hyperparameters and comparing model performance.

**Figure 6:**
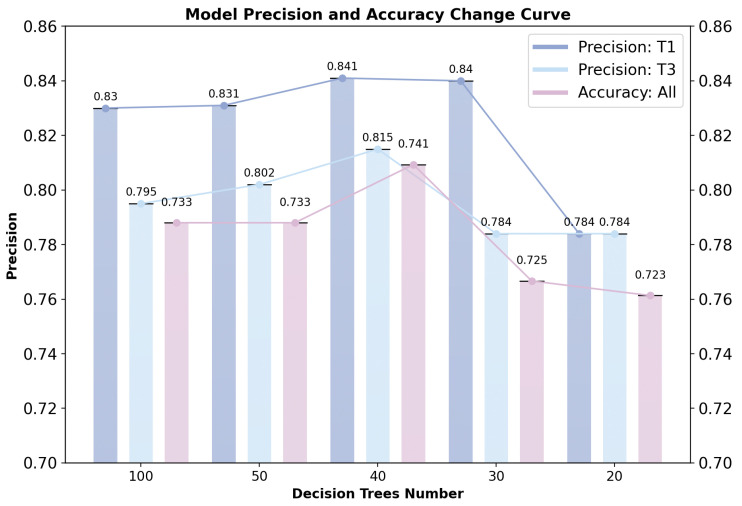
The changes in precision and comprehensive accuracy of T1 and T3 with the variation in the number of decision trees.

As the number of decision trees increases, both the precision for T1 and T3 and the overall accuracy initially increase, then decrease. When the number of decision trees reaches 40, the precision for T1 and T3 and the overall accuracy all reach their optimal values. Further increasing the number of decision trees has minimal effect on the model’s classification performance and may even lead to overfitting. Based on this analysis, the hyperparameter for the number of decision trees in the classification model is set to 40, with the training and testing dataset split at a ratio of 4:1. The specific classification results are shown in Figure 6.

(2) Hyperparameter Selection for the Second-Layer Random Forest Regression Model

**Figure 7:**
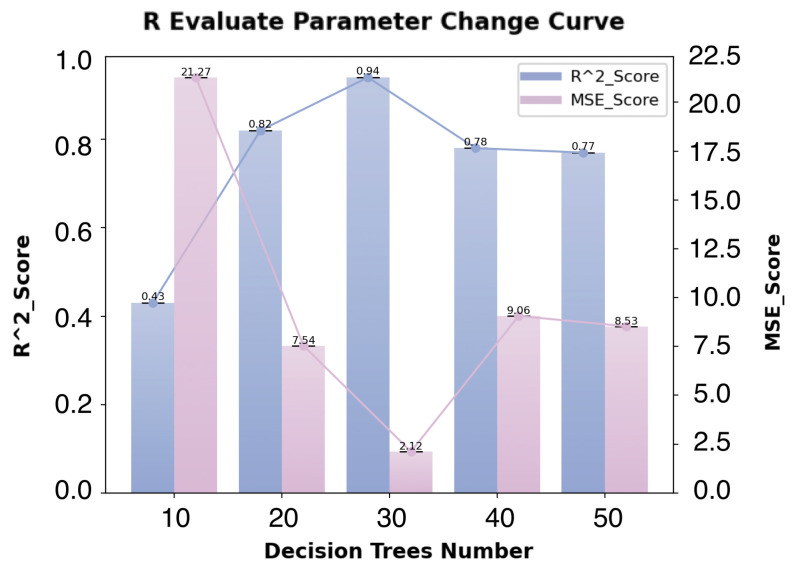
Change in $R^{2}$ and MSE error curve with number of decision trees in Random Forest regression.

In the Random Forest regression model, the $R^{2}$ evaluation parameter and the MSE error curve change with the number of decision trees, as shown in Figure 7.

As the number of decision trees increases from 10 to 30, the MSE error converges rapidly. When the number of decision trees reaches 30, the regression model’s convergence effect slows down, and the MSE and $R^{2}$ scores are optimized. Beyond this point, increasing the number of decision trees has little effect on improving model performance. Both MSE and $R^{2}$ remain relatively stable, and the quality of the model does not improve significantly.

Therefore, the optimal hyperparameter for the number of decision trees in the regression model is set to 30, as it results in the best balance of performance for MSE and $R^{2}$.

## 5. Results

This section presents the results of the first-layer Random Forest classification model and the second-layer Random Forest regression model.

### 5.1. First-Layer Random Forest Classification Model

(1) Classification Results

The average precision for the classification of the T1 (under-trust) and T3 (over-trust) segments is 82%. The recall rate for T1 is higher than its precision, indicating that the model performs better at identifying negative samples compared to positive samples. This suggests that the model’s accuracy in correctly identifying negative trust states (T1) exceeds its ability to correctly identify positive trust states (T3). The specific results are shown in Table 6.

**Figure 8:**
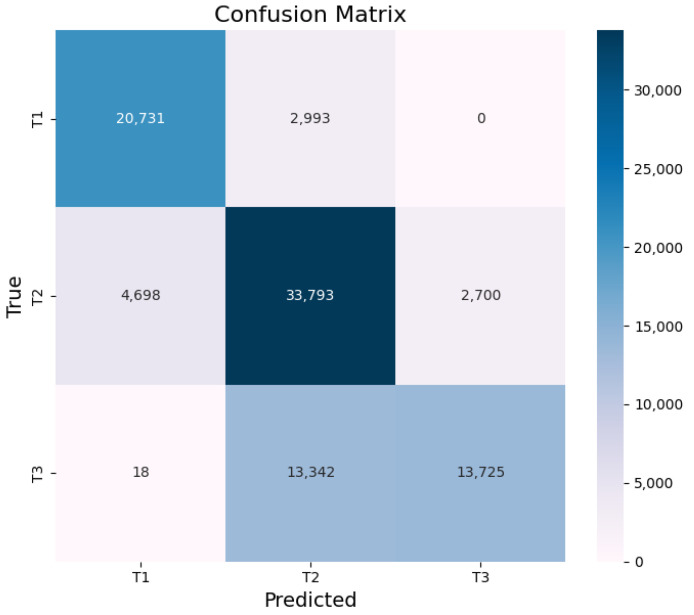
Confusion matrix analysis of model classification results.

Based on the confusion matrix analysis in Figure 8, the classification model demonstrates the best performance on T1 segments, with the amount of data misclassified into T3 segments being less than 0.0001 and into T2 segments being 0.12, resulting in a T1 recall rate of 88%. The model also performs well on T3 segments, with the amount of data misclassified into T1 segments being less than 0.001 and the amount classified correctly into T3 segments being 0.49, resulting in a T3 recall rate of 51%.

In summary, the classification model demonstrates excellent performance in distinguishing T1 and T3 segments, with an overall error rate of less than 0.1%. However, the model shows less distinction between T2 and T3 segments, resulting in a relatively low T3 recall rate. Subsequently, the eye-tracking data classified as T2 segments undergoes secondary fitting using the regression model.

(2) Importance of Eye-Tracking Metrics

**Figure 9:**
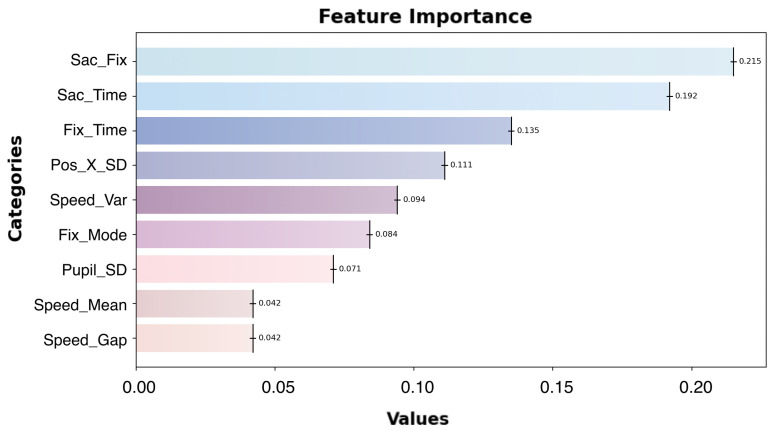
The importance ranking of the 9 oculomotor features output by the first-layer Random Forest classification model.

The importance of nine eye-tracking features was evaluated using the feature_importance method of the RandomForestRegressor, and their rankings are illustrated in Figure 9.

The summary of eye-tracking metrics is shown in Table 7. The features with the highest contribution are Sac_Fix (saccade–fixation ratio) and Sac_Time (saccade duration), with a combined importance exceeding 0.41. These metrics play a critical role in distinguishing between T1 (under-trust) and T3 (over-trust) states, highlighting their significance in trust calibration.

The next most influential features are Fix_Time (fixation duration) and Pos_X_SD (horizontal gaze dispersion), which contribute notably to the model’s performance. Fixation duration reflects focused attention, while horizontal gaze dispersion captures the breadth of horizontal search behavior, both of which are vital for understanding trust dynamics.

Following these are Speed_Var (saccade velocity variance), Fix_Mode (fixation mode), and Pupil_SD (pupil diameter standard deviation).

Finally, the features with the least impact are Speed_Gap (saccade velocity range) and Speed_Var (saccade velocity variance), each contributing less than 0.05, indicating a minimal role in the model’s predictive capability.

### 5.2. Second-Layer Random Forest Regression Model

(1) Feature Importance

**Figure 10:**
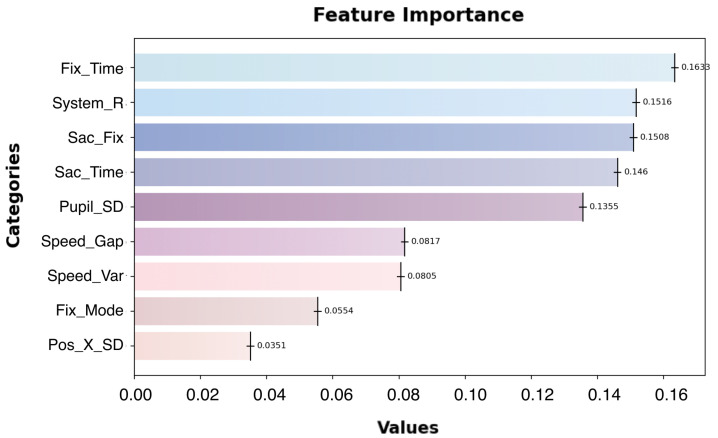
The importance ranking of the 9 oculomotor features output by the second-layer Random Forest regression model.

In this stage, T2 ambiguous trust segments were used as the dataset for the regression model. One eye-tracking feature, mean saccadic velocity, was removed, leaving seven other eye-tracking metrics. Additionally, a system reliability level metric was introduced, and the data were standardized. The contribution rates of the eye-tracking metrics in the second-layer Random Forest regression model are shown in Figure 10.

Fix_Time (fixation duration), Sac_Time (saccade duration), and Sac_Fix (saccade–fixation ratio), which are saccade–fixation-related metrics, contribute the most to trust state fitting, with a combined importance exceeding 0.45. These metrics highlight the critical role of attention dynamics in understanding and modeling trust states.

System reliability level ranks second in contribution, following the fixation and saccade metrics. This indicates that system reliability is a key environmental parameter in the evaluation and fitting of human–agent trust states, emphasizing its relevance in real-world trust calibration assessments.

(2) Model Fitting Results

The performance of the trust regression model was evaluated using the T2 segment eye-tracking dataset. The fitting results, including MSE, RMSE, and $R^{2}$, are presented in Table 8. The MSE and RMSE values indicate good accuracy in predicting trust levels. The coefficient of determination ($R^{2}$) is 0.8993, reflecting a strong fit of the model in capturing the variance of human–machine trust states.

## 6. Discussion

This study demonstrates that eye-tracking technology can be effectively used to identify operators’ different trust calibration states in automated supervisory control tasks. It highlights the correlation between eye-tracking features and human–agent trust states. Furthermore, a novel two-layer Random Forest model was introduced, establishing a trust calibration state classification and regression model based on eye-tracking features. The results showcase the high accuracy and reliability potential of eye-tracking features combined with hierarchical machine learning techniques in measuring trust calibration states.

### 6.1. Trust Measurement and Trust Calibration

Trust and trust calibration often coexist, and an increasing number of studies have recognized that merely increasing user trust is insufficient. It is crucial to achieve calibrated or appropriate trust levels. Measuring trust in a continuous, real time, and non-intrusive manner, and identifying whether users are in a state of over-trust or under-trust, is more beneficial for guiding system evaluation and design practices in real-world scenarios. This foundational idea serves as the starting point of our research.

Since the user-perceived scale of trust differs from the system’s capability measurement, the practical implementation of trust calibration requires careful exploration. Human–agent trust was modeled using the expected value of a Beta distribution with performance-inducing parameters. Missing scores of subjective trust scales were inferred based on an array constructed from real-time interaction data and system log data within the training dataset. To represent the user’s perception scale, the data were normalized and expressed on a 0–100 scale. Similarly, the system agent’s reliability was measured as the ratio of the sum of hits and correct rejections to the total number of tasks, with this ratio also normalized to a 0–100 scale. Using this approach, trust states were classified into under-trust, calibrated trust, and over-trust based on the comparison between user perception and system reliability.

Currently, three well-established strategies exist for measuring trust calibration: relative measures, correlational measures, and behavioral measures. The method proposed in this study falls under correlational measures, as it directly quantifies the relationship between system capability and perceived trustworthiness. This approach offers superior real-time performance and greater flexibility across application scenarios. Unlike relative measures [52], it provides explicit diagnostics for under-trust and over-trust states. Compared to behavioral measures [53], it is more suitable for real-time assessment and does not rely heavily on predefined behavioral standards, enhancing its applicability for system design and evaluation in varied contexts.

### 6.2. Contribution of Eye-Tracking Metrics to Trust Calibration in Human–Agent Interaction

In the final model results, saccade duration (Sac_Time), fixation duration (Fix_Time), and saccade–fixation time ratio (Sac_Fix) were identified as key contributors to the determination of human-agent trust states. These metrics highlight the critical role of attention allocation in determining trust calibration states. The balance between saccades and fixations provides a window into user trust behavior, allowing systems to dynamically assess and adapt to trust states. By leveraging these insights, designers can create systems that mitigate the risks of over-trust and under-trust, fostering more effective human-agent interactions.

The saccade–fixation ratio represents the proportion of time spent on saccades relative to the total time spent on both saccades and fixations. (1) A higher ratio suggests increased exploratory behavior, often reflecting under-trust, user uncertainty, or anxiety. Users actively scan the environment and question the system’s reliability, engaging in over-monitoring behaviors. (2) A lower ratio indicates focused attention on specific targets with reduced exploratory activity, often associated with over-trust or complacency, as users rely excessively on automation and neglect active monitoring.

The saccade duration is the total time spent on saccadic eye movements (shifts between fixations). (1) A longer saccade duration indicates active monitoring behavior and is often associated with under-trust, where users frequently shift their gaze to verify system actions or gather additional information. This is common in situations involving task complexity or uncertainty about system performance. (2) A shorter saccade duration reflects over-trust, where users allocate less time to scanning the environment, assuming the system can handle tasks reliably without their intervention.

The fixation duration is the time spent fixating on specific areas of interest (AOIs). (1) A longer fixation duration indicates concentrated attention and cognitive engagement, often reflecting confidence in the system’s reliability or task-related focus. Users dedicate more time to monitoring specific elements rather than scanning widely. (2) A shorter fixation duration suggests frequent scanning or monitoring, often linked to under-trust. Users may exhibit uncertainty about the system’s reliability and compensate with increased oversight. (3) A balanced fixation duration reflects calibrated trust, where users efficiently allocate attention between confirming system performance and exploring new information.

### 6.3. Limitations and Future Directions

This study has certain limitations that warrant further exploration.

(1) Theoretical Scope

From a theoretical perspective, this study strictly adheres to Lee and See’s definition of calibrated trust, which is described as “trust that matches the true capabilities of automation”. However, system capability (i.e., performance) is only one of the three key factors influencing trust. In addition to performance, system processes (how the system operates internally) and purposes (the intent behind the system’s design) also play critical roles in shaping the trust process. These aspects were not fully considered in this study.

(2) Practical Complexity

In real-world applications of complex information systems, tasks are often larger in number, more diverse in type, and may exhibit non-linear relationships between them. These factors pose additional challenges to the measurement of trust. Addressing these complexities and refining trust measurement methods for such systems is a crucial direction for future research.

Moving forward, expanding the framework to incorporate system processes and purposes, and testing the proposed method in more complex, dynamic environments, will be key to advancing trust measurement and calibration research.

## 7. Conclusions

To address the issues of intrusiveness, subjectivity, and intermittency in current trust measurement methods, as well as the specific characteristics of automated supervisory control tasks, this study proposes a novel trust calibration state recognition method based on eye-tracking data. Key eye-tracking metrics, including saccade duration, fixation duration, and the saccade–fixation ratio, demonstrating significant contributions to human–agent trust determination. This method not only provides a new approach to measuring human–agent trust but also offers strong support for the next step in designing adaptive interfaces.

## Figures and Tables

**Table 1:** The four scenarios of proxy monitoring outcomes.

True/Predicted	Alarms	No Alarms
Danger Exists	Hits	Misses
No Danger Exists	False Alarms	Correct Rejections

**Table 2:** Data collection formats for objective and subjective indicators.

No.	Interaction Data	System Data	Scale Data	Eye-Tracking Data
1	No	Hit	Complete the trust scale 1 time for each of the 6 subtasks on a scale of 1–100	Eye-tracker real-time collection of eye-tracking data throughout
2	Yes	Not Hit		
3	No	False Alarm		
4	Yes	Hit		
5	Yes	Correctly Reject		
6	No	Hit		

Repeat the above steps 5 times for a total of 30 completed tasks.

**Table 3:** Types of combinations between actual and predicted outcomes.

True/Predicted	T1	T2	T3
T1	T$P_{1}$	F$P_{1}$	F$P_{2}$
T2	F$N_{1}$	T$P_{2}$	F$P_{3}$
T3	F$N_{2}$	F$N_{3}$	T$P_{3}$

**Table 4:** RMSE metrics across models of varying reliability levels.

Reliability Level	RMSE
50%	0.0367
70%	0.0226
80%	0.0678
90%	0.0664
Comprehensive	0.0521

**Table 5:** Eye-tracking correlation analysis.

No.	Eye-Tracking Metric	Spearman Coefficient	Correlation Result
1	Fixation Duration Mean/Fixation Duration Standard Deviation	0.803	Strong correlation
2	Fixation Index	0.853	Strong correlation
3	Horizontal Search Range	0.663	Moderate correlation
4	Vertical Search Range	0.255	Weak correlation
5	Fixation Transition Time	0.859	Strong correlation
6	Fixation–Saccade Time Ratio	0.809	Strong correlation
7	Saccade Velocity Range	0.496	Moderate correlation
8	Saccade Velocity Variance	0.542	Moderate correlation
9	Saccade Velocity Mean	0.492	Moderate correlation
10	Pupil Diameter Change Rate	0.916	Strong correlation

**Table 6:** Human–agent trust discrimination model evaluation results.

Evaluation Metric	Results
T1 Precision	0.80
T3 Precision	0.84
T1 Recall	0.88
T3 Recall	0.51
T1 as T3 Error Rate	0.0000
T3 as T1 Error Rate	0.0006

**Table 7:** Eye-tracking metric summary.

Eye-Tracking Metric	Description
Sac_Fix (saccade–fixation ratio) Sac_Time (saccade time)	Proportion of time spent on saccades relative to total time spent on saccades and fixations. Total time spent on saccadic eye movements.
Fix_Time (fixation time)	Time spent fixing on specific areas of interest (AOIs).
Pos_X_SD (horizontal gaze dispersion)	Standard deviation of gaze positions along the horizontal axis.
Speed_Var (saccade velocity variance)	Variability in the velocity of saccadic eye movements.
Fix_Mode (fixation mode)	Most frequent fixation duration observed during a task.
Pupil_SD (pupil diameter standard deviation)	Standard deviation of pupil size changes during the task.
Speed_Gap (saccade velocity range)	Range between the maximum and minimum velocities of saccadic movements.
Speed_Mean (saccade velocity mean)	Average velocity of saccadic movements.

**Table 8:** The performance of the T2 fragment eye-tracking dataset within the second-layer Random Forest fitting model.

Criteria	Result
MSE	1.8439
RMSE	1.3579
$R^{2}$	0.8993

## Data Availability

Data are contained within the article.

## Appendix A. Final Scale

Based on previous work on the trust scale (Gulati 2019 [12]), adaptive descriptive adjustments were made according to the system under test. The revised scale consists of the following 12 items:

(1) I believe there could be negative consequences when using the system.
(2) I feel I must be cautious when using the system.
(3) It is risky to interact with the system.
(4) I believe the system will act in my best interest.
(5) I trust the system will do its best to assist me when I need help.
(6) I believe the system is genuinely interested in understanding my needs and preferences.
(7) I think the system is competent and effective in its functions.
(8) I believe the system performs its role very well.
(9) I trust that the system has all the functionalities I would expect from it.
(10) If I use the system, I feel I can depend on it completely.
(11) I can always rely on the system for support.
(12) I trust the information presented to me by the system.
